# Bone Regeneration: Overview and Future Trends

**DOI:** 10.3390/jcm12134529

**Published:** 2023-07-06

**Authors:** Nicola De Angelis, Francesco Bagnasco, Andrea Amaroli

**Affiliations:** 1 Unit of Implant and Prosthodontics, Department of Surgical Sciences and Integrated Diagnostics (DISC), University of Genoa, 16132 Genoa, Italy; fcbagna5@hotmail.it; 2 Faculty of Dentistry Department of Periodontology, Trisakti University, Jakarta 11440, Indonesia; 3 Faculty of Dentistry, Tunku Abdul Raman University (UTAR), Sungai Buloh 31900, Malaysia; 4 Department of Earth, Environmental and Life Sciences (DISTAV), University of Genoa, 16132 Genoa, Italy; andrea.amaroli@unige.it

It has been calculated that 8.6 million individuals in the US and European countries will show edentulism by the year 2050 [1], and this number will be much greater in developing countries. Subjects aged 35 to 45 may exhibit, per WHO guidelines [2], the maximum prevalence of partial edentulousness, and due to difficulties accessing dental treatment, the condition can evolve to total edentulousness in older people. Tooth loss, which can be connected to traumas, periodontal disease, traumatic extractions, and cancer of the mouth, may lead to moderate to severe bone deficiencies. A bone defect is defined as an anatomical condition that does not allow the tridimensional placement of implants [3]. In order to restore lost anatomy and function, alveolar bone augmentation is often required. Much progress has been made in recent decades, but several challenges still exist concerning hard tissue augmentation procedures. Bone substitutes and scaffolds are the main key materials for bone augmentation techniques.

The grafting material may be autogenous, derived from the same subject, or heterologous, derived from a different species. However, in recent decades, the use of self-transplant has been slowly reduced because of its invasiveness and augmented co-morbidity, despite its higher capacity to induce new bone formation [4]. Alternatively to immediate self-grafting, human bone can be retrieved from cadavers and treated with a rigorous deantigenation process to generate material with very high osteoconductive properties because of its similarity to the receiving area, reducing the invasiveness of the procedure. A recent randomized trial [5] compared the utilization of frozen radiation-sterilized allogenic bone grafts (FRSABG) and deproteinized bovine bone mineral (DBBM) in conjunction with a bioabsorbable collagen membrane for periodontal-guided tissue regeneration procedures.

Results showed that both materials can be successfully employed, but in terms of reducing the postoperative probing depth and linear defect fill, the allogenic graft significantly enhanced biological processes.

Another study [6] investigated the in vivo efficacy of a cancellous particulate allograft bone in the regeneration of post-extractive atrophic sites. Sites were grafted seven days after tooth extraction, and after 5 months, samples of the regenerated sites were taken contextually to implant insertion. The samples were histologically and histomorphometrically analyzed. Vertical and horizontal augmentation was successful, and in the sixth year of evaluation, the bone resorption around the implants was at 0.14 mm.

In a recent systematic review [7], the authors analyzed patients undergoing alveolar ridge preservation after tooth extraction to determine the grafting material that most effectively reduces horizontal and vertical ridge resorption compared to spontaneous healing. They also investigated which material promotes bone formation in the extraction sockets. The study included eighty-eight RCTs, involving a total of 2805 patients and 3073 sockets. Their comparison was made between xenografts (xg) and allografts (AG) used alone or in combination with bio-active agents (BIO+AG).

Network meta-analysis confirmed the consistency of XG for ridge dimension preservation, but several other materials and combinations, like AG, Bio + AG, and AG + alloplasts, produced even better results than XG in clinical comparisons. 

Implantation sites also represent different behaviors and regeneration capacities according to the remaining bone walls surrounding the defects. Moreover, the best results regarding material integration and new bone formation can be observed in sinus augmentations, due to the natural self-containing property of the surgically generated cavity.

A comprehensive review published in 2021 [8] examined the impact of residual bone height (RBH) and vertical bone gain on new bone formation (NBF) and graft shrinkage after lateral sinus lifts involving various biomaterials. The assessment encompassed graft volumetric changes, RBH, vertical bone gain, implant failure, and postoperative complications. The findings revealed that NBF was mostly unaffected by the preoperative bone height. Conversely, smaller graft volumes correlated with higher levels of new bone formation and reduced graft shrinkage. Minimizing the amount of augmentation could potentially contribute to improved graft healing and stability, particularly when utilizing alloplastic materials and xenografts.

In a recent retrospective investigation [9], researchers conducted a comparison between plant-based hydroxyapatite derived from algae (Algipore^®^ FRIOS^®^) and demineralized inorganic bovine bone (Bio-Oss^®^) when used alongside autologous PRP derived from the blood to elevate the sinus floor. Findings from clinical and radiographic assessments indicated a similar extent of newly generated bone in both cohorts, following seven years of functional loading for implants inserted after sinus augmentation employing permeable fluorohydroxyapatite and inorganic bovine bone. No notable disparity in the marginal bone loss was observed around the implants within either group. Additionally, post-extraction sites exhibited great potential in terms of regeneration because of the anatomy of the residual defect, and showed even greater promise when different systems of sealing were applied.

Regarding this subject, a comprehensive review conducted in 2021 [10] explored whether the placement of a biomaterial over an extraction socket yields improved outcomes concerning both horizontal and vertical alveolar dimensional changes, as well as the percentage of new bone formation, in comparison to socket healing without coverage. The study also aimed to determine which biomaterial demonstrates superior results. The analysis encompassed twelve trials and assessed a total of 312 sites. Regarding horizontal changes, autologous soft tissue grafts exhibited better outcomes than resorbable membranes. Compared to having no membrane, a statistically significant difference in favor of resorbable membranes was identified, showing no significant heterogeneity. Moreover, a statistically significant difference favoring non-crosslinked membranes was found compared to crosslinked membranes, which was further supported by histomorphometric meta-analysis. In conclusion, the coverage seems to enhance new bone formation, but no recommendations can be given on which system provides better results, and similar results were retrieved later in a preliminary report [11].

An excellent bone formation, as preservation of the extraction socket volume, is also a key factor for soft tissue enhancement (keratinized tissue width and high). In 2022 a systematic review [12] focused the investigation on which type of sealing system and graft is able to provide better soft tissue outcomes. Grafting materials exhibited statistically significant improvements in soft tissue thickness and vertical buccal height changes when utilized in conjunction with crosslinked collagen membranes. Conversely, soft tissue grafts yielded superior results regarding horizontal width changes. Non-crosslinked membranes and other materials or combinations demonstrated slightly less favorable outcomes.

Sealing systems can simply be barriers to contain the grafting material and guide the soft tissue closure, or be derived from autogenous blood and therefore also be carriers of growth factors, such as Platelet-Derived Growth Factor (PDGF), and/or be infused like grafts by the operator with specific proteins involved in bone regeneration (Bone Morphogenetic Proteins, BMPs) [13]. Autogenous blood-derived PRP or PRF is often employed as a membrane onto the material, as described in a prospective clinical study published in 2022 [14].

More recently, customized devices/systems have also been investigated to shorten surgical times, augment precision and adaptation, and ensure the perfect fitting of the graft and/or the barrier. In a case series study [15], the authors described the advantage of customized titanium meshes for rehabilitating severe atrophic ridges. These devices were designed with CAD software with 3D imaging from the patient and then printed by a Selective Laser Melting (SLM) system from titanium alloy powder. Despite the advantages of precision and adaptation, the authors also reported quite a high incidence of exposures during the healing phase.

Similar findings were also retrieved in a recent systematic review [16], where the incidence of the exposures for customized titanium meshes was almost 20%, but none of the included randomized trials described the jeopardizing of consequent implant placement.

The use of customized devices/grafts surely solves the surgical problems related to adaptation, fitting, and precision. However, it still does not present a solution that can minimize surgical interventions and enlarge the availability of products to address unequal access to bone augmentation procedures.

Therefore, the aim for future research and clinical studies should address the use of the largely available material, such as resorbable biopolymers, that can be easily printed by the Fused Deposition Modeling technique (FDM) [17]. In the mentioned study, the authors showed the capacity of ceramic biopolymers in terms of cell proliferation and maturation, which is one of the most important milestones for medical grade classification. Moreover, after identifying the proper material, the possibility to “self-print” the devices would greatly contribute to tackling the inequalities in access to dental care, especially in developing countries.
